# Jet Electrochemical Micromilling of Ti-6Al-4V Using NaCl–Ethylene Glycol Electrolyte

**DOI:** 10.3390/mi15020173

**Published:** 2024-01-24

**Authors:** Shen Niu, Kaiqiang Huang, Pingmei Ming, Siru Wang, Fei Zhao, Ge Qin, Huan Liu

**Affiliations:** 1 School of Mechanical and Power Engineering, Henan Polytechnic University, Jiaozuo 454003, China; hkq15638019737@163.com (K.H.); mingpingmei@163.com (P.M.); 18855033057@163.com (S.W.); qinge@hpu.edu.cn (G.Q.); liuhuan@hpu.edu.cn (H.L.); 2 College of Safety Science and Engineering, Henan Polytechnic University, Jiaozuo 454003, China; zhaofei@hpu.edu.cn

**Keywords:** jet electrochemical micromilling, titanium alloy, NaCl–ethylene glycol electrolyte, stray corrosion, multiple passes

## Abstract

Titanium alloys are widely used in aerospace and biomedicine because of their excellent mechanical characteristics, but these properties also make such alloys difficult to cut. Jet electrochemical micromilling (JEMM) is based on the principle of electrochemical anodic dissolution; it has some inherent advantages for the machining of titanium alloy microstructures. However, titanium oxidizes readily, forming an oxide film that impedes a uniform dissolution during electrochemical machining. Therefore, a high voltage and an aqueous NaCl electrolyte are usually used to break the oxide film, which can lead to severe stray corrosion. To overcome this problem, the present study investigated the JEMM of Ti-6Al-4V using a NaCl–ethylene glycol (NaCl-EG) electrolyte. Electrochemical testing showed that Ti-6Al-4V exhibits a better corrosion resistance in the NaCl-EG electrolyte compared to the aqueous NaCl electrolyte, thereby reducing stray corrosion. The localization and surface quality of the grooves were enhanced significantly when using JEMM with a NaCl-EG electrolyte. A multiple-pass strategy was adopted during JEMM to improve the aspect ratio, and the effects of the feed depth and number of passes on the multiple-pass machining performance were investigated. Ultimately, a square annular microstructure with a high geometric dimensional consistency and a smooth surface was obtained via JEMM with multiple passes using the optimal parameters.

## 1. Introduction

Titanium alloys have a high specific strength, exceptional biocompatibility, outstanding corrosion resistance, and an impressive performance at both high and low temperatures, thereby demonstrating considerable potential for applications in metallic microfabrication [1,2,3]. In the past decade, titanium alloys have been used increasingly in aerospace, biomedicine, and precision engineering, and a pressing necessity for advancing these high-end industries involves manufacturing critical microscale components such as microgrooves, microcavities, micropump impellers, and miniature medical implants [4,5,6]. However, titanium alloys have a low elastic modulus, poor thermal conductivity, and high chemical reactivity, which makes them extremely difficult to cut. Therefore, high-efficiency and high-quality micromachining of titanium alloy microcomponents is a significant challenge for present microfabrication technologies.

Jet electrochemical micromilling (JEMM) is an electrochemical machining (ECM) method. It employs a metal nozzle with an outer diameter less than 1 mm as the tool cathode and is combined with high-precision multi-axis numerical control movements [7]. JEMM is based on electrochemical anodic dissolution for processing metallic materials; the noncontact machining process avoids some of the drawbacks associated with traditional mechanical machining, such as rapid tool wear, the formation of burrs, and deformation [8,9]. The low temperature in the machining area prevents thermal damage to the machined surface, such as recast layers, heat-affected zones, and microcracks caused by laser processing and electrical discharge machining, which can compromise the operational performance [10,11]. In addition, the advantages of JEMM also include a high machining flexibility, no surface stress, and independence from workpiece hardness [12]. Therefore, JEMM shows promise as a better process for high-efficiency, high-quality micromachining of difficult-to-cut materials for complex microcomponents.

Hackert-Oschätzchen et al. [13,14] researched JEMM using a metal nozzle; by controlling the movement path of the metal nozzle and the processing parameters, microcavities with a depth of 180 µm, a width of 190 µm, and a surface roughness (*Ra*) of 0.1 µm were machined on stainless steel, and internal convex structures with a maximum depth of 167 µm and a minimum depth of 8 µm were fabricated on tungsten carbide. Clare et al. [15,16] proposed a JEMM method involving a metal nozzle with an adjustable jet angle; it was found that having the electrolyte jet direction form an acute angle with the feed direction resulted in a significantly lower surface roughness, and typical biomedical stent geometries were machined on nickel-based superalloys using this method. Guo et al. [17,18,19] researched coaxial aspirated JEMM of stainless steel and plastic mold steel; by rapidly removing the electrolyte outside the central region of the metal-nozzle jet, the stray corrosion on the machined surface of the workpiece was effectively reduced, and machining localization was improved. Ming et al. [20,21] researched kerosene-submerged horizontal JEMM of stainless steel; the results indicated that compared to having a vertical metal-nozzle jet, a horizontal one reduced the contact area between the reflected electrolyte and the outer wall of the metal nozzle, and this also increased the thickness of the electrolyte flow film and accelerated the removal of electrolytic products, thereby offering significantly enhanced machining localization. Hung et al. [22] subjected stainless steel to JEMM using a metal nozzle; they showed that using a mixed-gas electrochemical jet can reduce the stray current in the nonprocessing zone of the workpiece, and they produced microscale blind grooves with a surface roughness (*Ra*) of 0.03 µm under a feed rate of 1.68 mm/s and gas and electrolyte inlet pressures of 0.1 MPa.

However, JEMM research to date has been focused mainly on stainless steel, tungsten carbide, mold steel and nickel-based superalloys, with few investigations involving titanium alloys. This is because during the ECM of titanium, a dense oxide film forms on the surface, thereby hampering steady dissolution. Therefore, a high voltage and aqueous NaCl electrolytes are usually used in ECM to break this oxide film. Cao et al. [23] found that the active Cl^−^ ions in an aqueous NaCl solution have a strong ability to break the oxide film on Ti-6Al-4V, and a grid-like convex structure was obtained on a cylindrical titanium alloy workpiece with a diameter of 90 mm. Xue and Qu [24] used a high voltage of 50 V to create grooves with a width of 10 mm via macro-electrolyte jet machining. However, a high voltage and an aqueous NaCl electrolyte led to severe stray corrosion during ECM, which resulted in a poor machining accuracy and surface quality [23,24,25], and so these conditions are unsuitable for the JEMM of titanium alloy microstructures.

In this paper, NaCl–ethylene glycol (NaCl-EG) electrolyte is proposed in the JEMM of Ti-6Al-4V to reduce stray corrosion. The results of polarization curves and electrochemical impedance spectroscopy (EIS) of Ti-6Al-4V in aqueous NaCl electrolyte and NaCl-EG electrolyte are compared and analyzed. Furthermore, a multiple pass strategy is adopted to improve the aspect ratio in the JEMM of Ti-6Al-4V using the NaCl-EG electrolyte. The effects of the feed depth and number of passes on the aspect ratio and stray corrosion of JEMM with multiple passes are investigated. Finally, a Ti-6Al-4V square annular microstructure is produced with the optimal parameters via JEMM with multiple passes using the NaCl-EG electrolyte.

## 2. Materials and Methods

### 2.1. Materials and Electrolytes

Ethylene glycol is a colorless, odorless liquid organic compound with the properties as given in Table 1. With a high boiling point, suitable viscosity, and the ability to dissolve moderate amounts of NaCl, it can achieve a good electrical conductivity, meeting the solvent requirements for JEMM. Using ethylene glycol as a solvent and NaCl as a solute produces the NaCl-EG electrolyte. The titanium alloy used in this study was Ti-6Al-4V, an α + β titanium alloy that is used widely in various applications, and its main components are given in Table 2.

### 2.2. Experimental System and Arrangement

**Figure 1:**
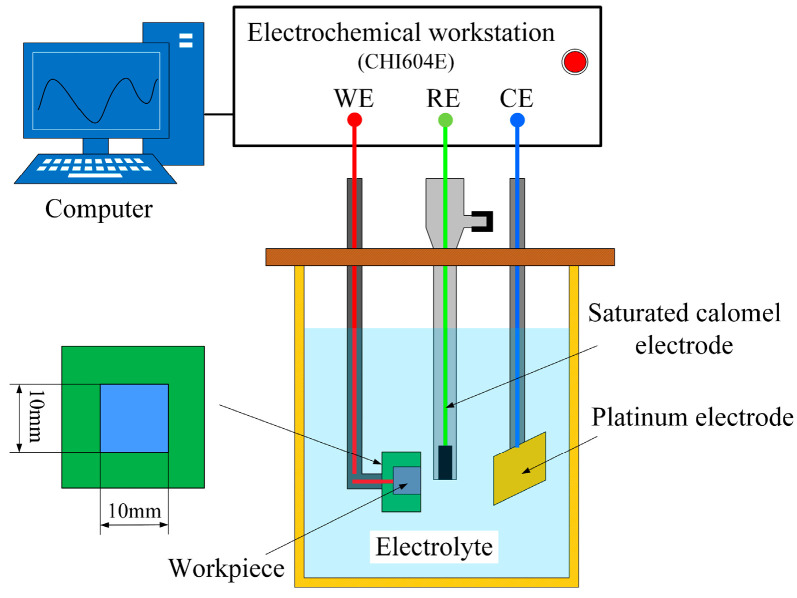
Schematic of the device used to measure polarization curves and EIS.

Electrochemical testing is generally used to reveal the mechanism and regularities of electrochemical behavior during ECM [26]. In the present study, Ti-6Al-4V was subjected to electrochemical testing (including polarization curves and EIS) in the aqueous NaCl electrolyte and NaCl-EG electrolyte. This testing was performed using a three-electrode system in an electrochemical workstation (CHI604E; CH Instruments, Shanghai, China). As shown in Figure 1, the system had a platinum electrode as the counter electrode, a saturated calomel electrode as the reference electrode, and the Ti-6Al-4V specimen (10 mm × 10 mm × 10 mm) as the working electrode. The Ti-6Al-4V specimen was fixed in a bespoke epoxy-resin fixture, with only 1 cm^2^ of its surface exposed to the electrolyte; before the experiments, the surface to be measured was ground gradually from 240 to 2000 grit using SiC paper and then cleaned ultrasonically in acetone.

EIS and polarization curve measurements were carried out separately in 1 mol/L aqueous NaCl electrolyte and 1 mol/L NaCl-EG electrolyte. The EIS measurements were carried out at the open circuit potential using a signal with a perturbation of 5 mV in the alternating current (AC) frequency ranges of 10^5^–10^−1^ Hz in aqueous NaCl electrolyte and 10^5^–10^−2^ Hz in NaCl-EG electrolyte. Subsequently, the polarization curve measurements were determined in the potential range of −1 V to 8 V at a sweep rate of 10 mV/s, and the measurements were stopped when the potential reached 8 V. Pre-polarization was performed before measuring the curves to remove any oxide film formed in the air. The EIS and polarization curve measurements were repeated three times for each set to ensure reproducibility.

**Figure 2:**
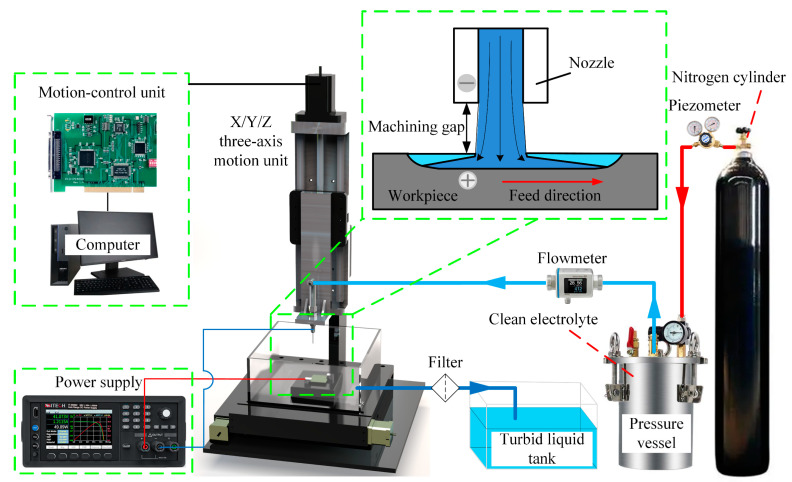
Schematic of jet electrochemical micromilling (JEMM) device.

As shown in Figure 2, the JEMM machining system used in this study comprised an X/Y/Z three-axis motion unit, a motion-control unit, an electrolyte supply unit, and a power supply. The workpiece was fixed to a specially designed worktable, allowing current transfer to the workpiece. The workpiece movement in the XY direction was achieved via the X/Y/Z three-axis motion unit, traveling along the machining path set by the motion control unit at the required feed rate during processing. The nozzle could move along the Z-axis to enable vertical feed. The nitrogen cylinder provided a stable pressure for the pressure vessel, ensuring that the electrolyte was supplied steadily to the machining area at the required pressure by the electrolyte supply unit. The tool cathode used in the experiments was a cylindrical hollow stainless-steel nozzle with an inner diameter of 300 µm and an outer diameter of 600 µm. The power supply (IT-N6952; ITECH, Nanjing, China) provided the required machining voltage, with its positive pole connected to the workpiece and its negative pole connected to the metal nozzle.

The experimental conditions are given in Table 3. Grooves were machined on Ti-6Al-4V in 1 mol/L aqueous NaCl electrolyte and 1 mol/L NaCl-EG electrolyte, and while machining a groove, the feed distance of the workpiece was set at 3000 µm. The three-dimensional morphology of the grooves was measured via laser confocal microscopy (OLS5100; Olympus, Tokyo, Japan).

**Figure 3:**
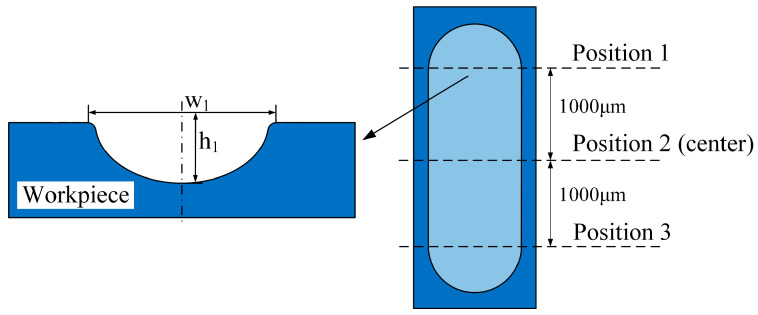
Schematic of performance evaluation indices.

The performance evaluation criteria used in the experiments were the average groove width *W*_0_, the average groove depth *H*_0_, and the aspect ratio *A*. As shown in Figure 3, the groove width and depth were measured at center position 2 and at positions 1 and 3 at 1000 µm from the groove center. The average groove width is

*W*_0_ = (*W*_1_ + *W*_2_ + *W*_3_)/3, (1)

where *W*_1_, *W*_2_, and *W*_3_ are the groove widths (µm) measured at positions 1, 2, and 3, respectively. The average groove depth is

*H*_0_ = (*h*_1_ + *h*_2_ + *h*_3_)/3, (2)

where *h*_1_, *h*_2_, and *h*_3_ are the groove depths (µm) measured at positions 1, 2, and 3, respectively. Finally, the aspect ratio is

*A* = *H*_0_/*W*_0_. (3)

## 3. Results and Discussion

### 3.1. Polarization Curves

**Figure 4:**
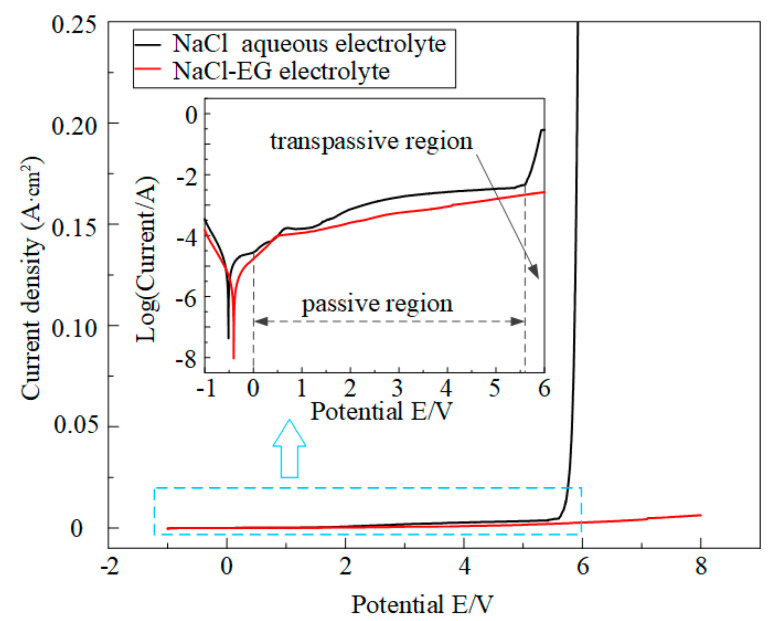
Polarization curves of Ti-6Al-4V in the aqueous NaCl electrolyte and NaCl-EG electrolyte.

Figure 4 shows the polarization curves of Ti-6Al-4V in the aqueous NaCl electrolyte and NaCl-EG electrolyte. In the aqueous NaCl electrolyte, Ti-6Al-4V exhibits obvious passive and transpassive regions: the workpiece enters the passive region when the potential is close to zero, and the current density barely increases with increasing potential; when the potential increases to 5.6 V, the workpiece enters the transpassive region, the oxide film is destroyed, and the current density increases sharply with increasing potential. In contrast, in the NaCl-EG electrolyte, Ti-6Al-4V does not exhibit distinct passive and transpassive regions, and the current density is much lower. The reason for these differences in the polarization curves is that in the NaCl-EG electrolyte, the generation rate of the oxide film is low during the anodization process because of the lack of water. The corrosion potential (*E*_corr_) and current density (*J*_corr_) obtained from the polarization curves are given in Table 4. For Ti-6Al-4V in the NaCl-EG electrolyte, *E*_corr_ is more positive than that in aqueous NaCl electrolyte; a more positive *E*_corr_ indicates better corrosion resistance, and so Ti-6Al-4V has a better corrosion resistance in the NaCl-EG electrolyte. According to Faraday’s laws, the corrosion rate is proportional to *J*_corr_, and the lower *J*_corr_ of Ti-6Al-4V in the NaCl-EG electrolyte indicates a lower corrosion rate. As discussed above, in the NaCl-EG electrolyte, Ti-6Al-4V does not exhibit distinct passive and transpassive regions, and the current density is lower than that in the aqueous NaCl electrolyte. Also, Ti-6Al-4V has a superior corrosion resistance in the NaCl-EG electrolyte, and thereby can reduce stray corrosion under stray currents in the non-machining area.

### 3.2. Electrochemical Impedance Spectroscopy

**Figure 5:**
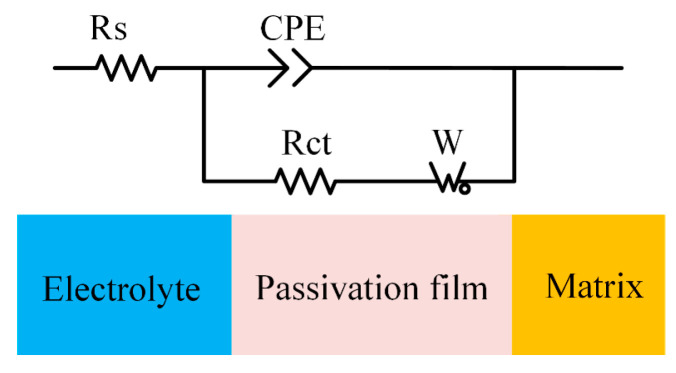
EEC model for fitting EIS data.

**Figure 6:**
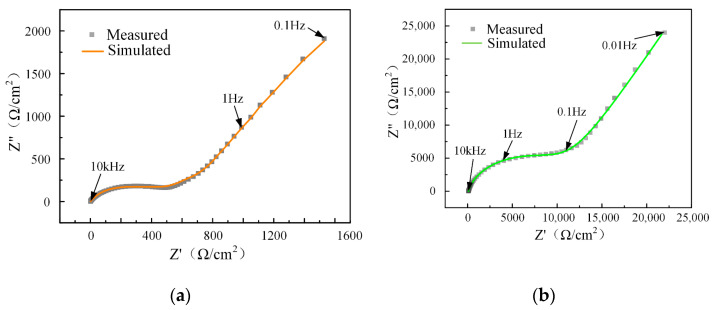
Nyquist plots for Ti-6Al-4V in (**a**) aqueous NaCl electrolyte and (**b**) NaCl-EG electrolyte.

As shown in Figure 5, the EIS data were fitted using the electrical equivalent circuit (EEC) model, including the electrolyte resistance (*R*_S_), the charge-transfer resistance (*R*_ct_), the Warburg impedance (*W*), and the constant phase element (CPE) to describe the double-layer capacitance. Comparing the Nyquist plots for Ti-6Al-4V in the two electrolytes in Figure 6, the curvature of the high-frequency region in the NaCl-EG electrolyte is significantly greater than that in the aqueous NaCl electrolyte. The curvature of the Nyquist plot in the high-frequency range reflects the influence of *R*_ct_ on the material’s surface [27], and the greater curvature in this frequency range indicates that the electrochemical reactions become more difficult with increasing *R*_ct_ [28]. The fitting results in Table 5 show that the *R*_ct_ of Ti-6Al-4V in the NaCl-EG electrolyte is significantly higher than that in the aqueous NaCl electrolyte, indicating that Ti-6Al-4V has a better corrosion resistance in the NaCl-EG electrolyte. Moreover, a larger *R*_S_ indicates a higher electrolyte resistance; thus, the stray current in the non-machining area is smaller, thereby reducing stray corrosion. Thus, Ti-6Al-4V has a superior corrosion resistance in the NaCl-EG electrolyte, thereby protecting it against stray corrosion in the non-machining area. This also explains why the polarization curves in the NaCl-EG electrolyte exhibit a more positive *E*_corr_.

### 3.3. JEMM of Ti-6Al-4V

**Figure 7:**
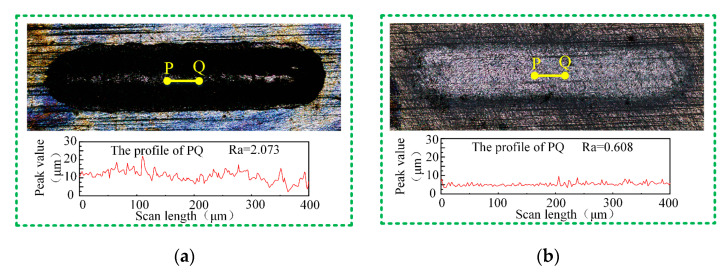
Grooves processed using (**a**) aqueous NaCl electrolyte and (**b**) NaCl-EG electrolyte.

Figure 7 shows grooves machined on Ti-6Al-4V in the aqueous NaCl electrolyte and the NaCl-EG electrolyte as visualized using laser confocal microscopy. Compared with using the aqueous NaCl electrolyte, the groove edges are straighter when using the NaCl-EG electrolyte. Also, the profile of the yellow line segment in the middle of each groove was measured, and the bottom profile of the groove machined in aqueous NaCl electrolyte exhibits noticeable peaks and valleys, indicating significant fluctuations. The calculated surface roughness for this segment is 2.112 ± 0.039 µm in the aqueous NaCl electrolyte and 0.605 ± 0.011 µm in the NaCl-EG electrolyte, indicating that the groove bottom is smoother in the NaCl-EG electrolyte.

**Figure 8:**
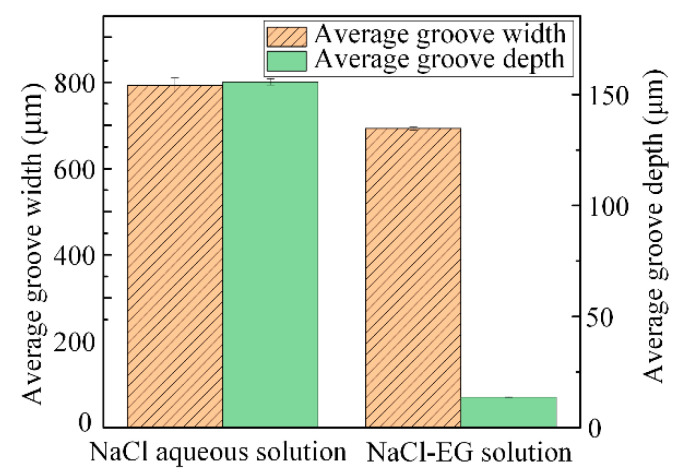
Machining performance for grooves machined using different electrolytes.

Figure 8 shows the groove width and depth as machined in each electrolyte. The widths of the grooves machined in the aqueous NaCl and NaCl-EG electrolytes were 793.393 ± 16.730 µm and 692.889 ± 4.299 µm, respectively. However, the groove depth machined with a single pass in NaCl-EG electrolyte was 13.646 ± 0.075 µm, while 155.721 ± 1.411 µm was achieved in the aqueous NaCl electrolyte. Therefore, the aspect ratio of the groove machined in the NaCl-EG electrolyte was smaller than that in the aqueous NaCl electrolyte. As discussed above, compared with the aqueous NaCl electrolyte, when using the NaCl-EG electrolyte, the groove was narrower, which indicates higher machining localization could be obtained by using the NaCl-EG electrolyte; however, the groove was shallower, which indicates that less material was removed in the depth direction by a single pass. These phenomena can also be correlated with the results of electrochemical testing. Compared to the aqueous NaCl electrolyte, Ti-6Al-4V has a superior corrosion resistance in NaCl-EG electrolyte, and this helps to reduce stray corrosion in the non-machined area, which results in better localization and surface quality. However, the lower dissolution rate also leads to a significantly reduced amount of material removal in the depth direction.

**Figure 9:**
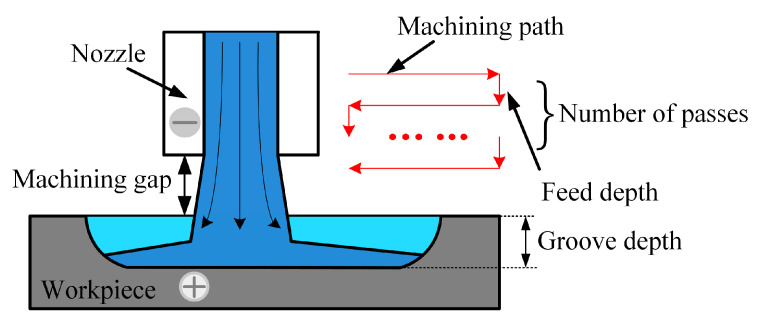
Schematic of multiple passes.

Although better machining localization and surface quality were obtained in the JEMM of Ti-6Al-4V by using the NaCl-EG electrolyte, the amount of material removed in the depth direction was very small in a single pass, which led to a significantly low aspect ratio. Therefore, multiple passes were used in JEMM to enhance the aspect ratio, and the machining process is shown in Figure 9. After completing one pass, the nozzle was fed downward by a certain depth, which is referred to as the feed depth. Then, the nozzle was passed over the workpiece again. The workpiece was continuously milled layer by layer along the machining path, until the entire machining process was completed. How the feed depth and number of passes affect the multiple-pass groove machining performance is investigated below.

#### 3.3.1. Effects of Feed Depth

Here, how the feed depth affects JEMM of Ti-6Al-4V with multiple passes is investigated. In the experiment, 1 mol/L of the NaCl-EG electrolyte was used, the nozzle feed distance was 3000 µm, eight passes were used, and the feed depth was zero, 10 µm, 20 µm, or 30 µm, with the other parameters as given in Table 3.

**Figure 10:**
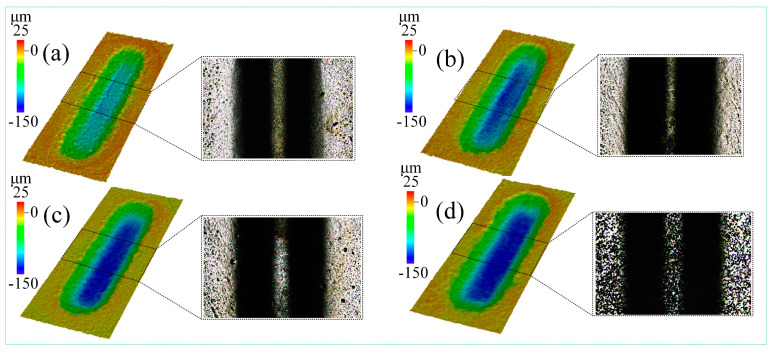
Morphology of grooves processed with a feed depth of (**a**) zero, (**b**) 10 µm, (**c**) 20 µm, and (**d**) 30 µm.

Figure 10 shows the morphology of the grooves processed with different feed depths as acquired using laser confocal microscopy. As can be seen, the groove depth and width are uniform and increase with an increasing feed depth. At a feed depth of 30 µm, significant stray corrosion occurs at the groove edges; a possible reason for this is that the machining gap between the nozzle and groove edges is reduced with increasing feed depth, which results in a large current density and material removal rate. Thus, the groove width and depth are both increased. Also, at a feed depth of 30 µm, the stray current at the groove edges is significantly increased, which leads to stray corrosion. Therefore, selecting appropriate processing parameters is important for reducing stray corrosion.

**Figure 11:**
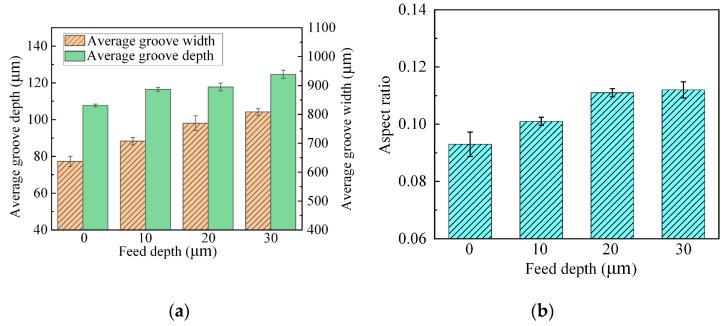
Machining performance for grooves machined with different feed depths: (**a**) average depth and width; (**b**) aspect ratio.

Figure 11 shows the machining performance for grooves machined with different feed depths. As shown in Figure 11a, when the feed depth was increased from zero to 30 µm, the groove depth increased from 77 µm to 104 µm and the width increased from 830 µm to 938 µm. As shown in Figure 11b, the aspect ratio also increased in general with increasing feed depth, but when the feed depth was increased from 20 µm to 30 µm, the aspect ratio barely increased. The increase in aspect ratio is attributed to the good localization of JEMM, which results in the dissolution rate in the depth direction being higher than that in the width direction. With a feed depth of 30 µm, the current density at the groove edges increased sharply, and the dissolution rate in the width direction increased. Consequently, the increase in aspect ratio was no longer significant. Based on the above discussion, the aspect ratio increases in general with increasing feed depth, but an excessive feed depth leads to an insignificant increase in the aspect ratio and stray corrosion at the groove edges. Ultimately, 20 µm was taken as the feed depth for JEMM of Ti-6Al-4V with multiple passes.

#### 3.3.2. Effects of Number of Passes

Here, how the number of passes affects the JEMM of Ti-6Al-4V with multiple passes is investigated. In the experiment, 1 mol/L of the NaCl-EG electrolyte was used, the nozzle feed distance was 3000 µm, the feed depth was 20 µm, and 6, 8, 10, or 12 passes were used, with the other parameters as given in Table 3.

**Figure 12:**
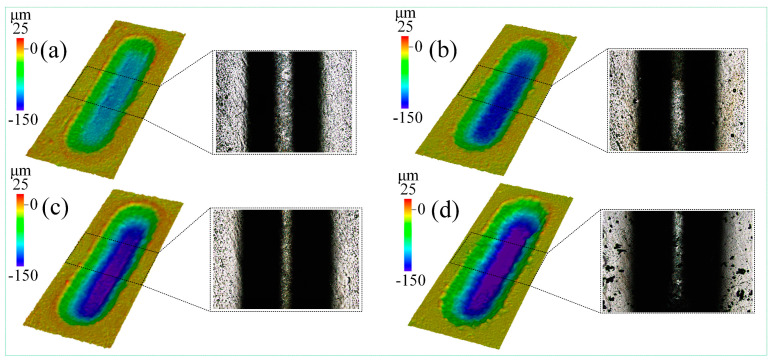
Morphology of grooves processed with (**a**) 6, (**b**) 8, (**c**) 10, and (**d**) 12 passes.

Figure 12 shows the morphology of the grooves processed with different numbers of passes as obtained using laser confocal microscopy. With more passes, the groove depth and width both increased. With 12 passes, there was stray corrosion at the groove edges. The main reason for this is that each pass in JEMM with multiple passes is accompanied by a certain amount of material removal; with more passes, more material is removed, and with 12 passes, the gap between the nozzle and groove edges narrows, so the stray current at the groove edges is large, which results in significant stray corrosion in that region.

**Figure 13:**
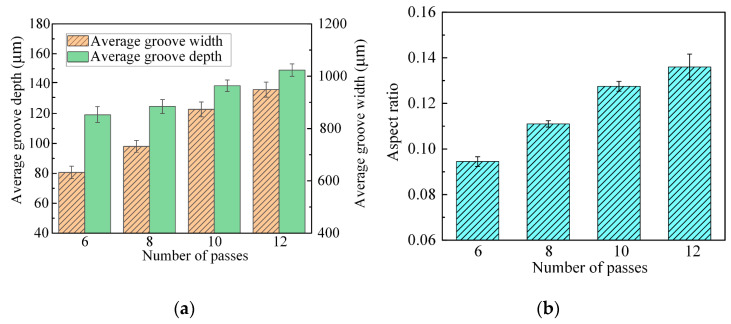
Machining performance for grooves machined with different numbers of passes: (**a**) average depth and width; (**b**) aspect ratio.

Figure 13 shows the machining performance for grooves machined with different numbers of passes. As shown in Figure 13a, when the number of passes was increased from 6 to 12, the groove depth increased from 80 µm to 136 µm and the width increased from 852 µm to 1023 µm. As shown in Figure 13b, the groove aspect ratio increased linearly with more passes, and this is attributed to the good localization achieved by JEMM, leading to a higher dissolution rate in the depth direction compared to that in the width direction, thereby resulting in a higher dissolution rate in the depth direction than in the width direction. Based on the above discussion, using more passes increases the groove aspect ratio in general, but too many passes may result in stray corrosion at the groove edges. As discussed above, the aspect ratio increases in general with an increasing feed depth, but excessive feed depth leads to significant stray corrosion at the groove edges. Therefore, only an appropriate number of passes can result in an increased aspect ratio and better surface quality. Ultimately, 10 passes were selected for JEMM of Ti-6Al-4V with multiple passes.

#### 3.3.3. Machining of a Microstructure

**Figure 14:**
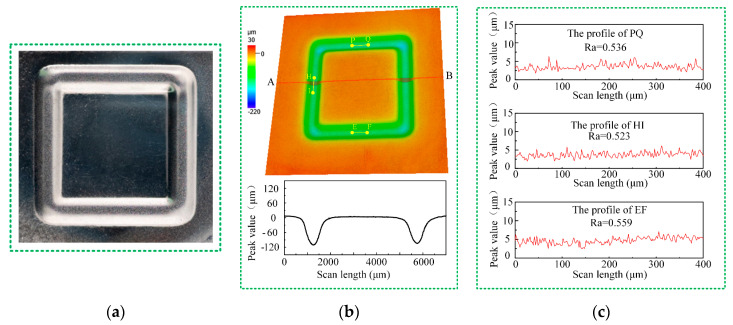
Square annular microstructure: (**a**) photograph; (**b**) morphology; (**c**) roughness.

Finally, a square annular microstructure was created on Ti-6Al-4V via JEMM with multiple passes using the NaCl-EG electrolyte, a feed depth of 20 µm, and 10 passes, with the other parameters as given in Table 3. By setting the machining path, the nozzle changed its feed direction every 4000 µm of travel, and the feed direction changed three times and this the nozzle moved a distance of 16,000 µm during each pass. Figure 14a shows a photograph of the microstructure as obtained using a camera (Z5; Nikon, Tokyo, Japan); as can be seen, the edges of the fabricated microstructure are straight, and the workpiece surface and machined area have smooth surfaces with a typical metallic luster. As shown in Figure 14b, the three-dimensional morphology of the microstructure as obtained via laser confocal microscopy reveals well-formed grooves with a high geometric dimensional consistency. From measuring the profile in the red cross-section AB, the results indicate groove widths of 121.9 µm and 117.6 µm and depths of 961.4 µm and 956.6 µm. Figure 14c shows the cross-sectional profiles of the groove bottoms in the three distinct regions shown in Figure 14b, as measured using laser confocal microscopy. As can be seen, the surface profiles of the microstructure are smooth, with few peaks or valleys. The measured surface roughness in the three regions of the microstructure is 0.536 µm, 0.523 µm, and 0.559 µm, and the calculated average width, groove depth, and roughness of the microstructure are 959 ± 3.4 µm, 119.8 ± 3 µm, and 0.539 ± 0.015 µm, respectively, which demonstrates the high geometric dimensional consistency in the fabricated microstructure. Therefore, JEMM with multiple passes using a NaCl-EG electrolyte offers enhanced machining localization, geometric dimensional consistency, and surface quality for Ti-6Al-4V. These results demonstrate the powerful capability of JEMM with multiple passes using NaCl-EG for fabricating titanium alloy complex microstructures with a high machining accuracy and a high surface quality.

## 4. Conclusions

Herein, the polarization curves and EIS results for Ti-6Al-4V in aqueous NaCl electrolyte and NaCl-EG electrolyte were compared and analyzed, and the effect of the feed depth and number of passes on the machining performance of JEMM with multiple passes using the NaCl-EG electrolyte was investigated. Finally, a square annular microstructure was obtained on Ti-6Al-4V via JEMM with multiple passes using the optimal parameters. The results led to the following conclusions.

1. The results of electrochemical testing indicate that Ti-6Al-4V has a better corrosion resistance in the NaCl-EG electrolyte, which leads to reduced stray corrosion in the non-machined regions. The machining localization and surface quality of the grooves machined by JEMM using the NaCl-EG electrolyte were better than those in the aqueous NaCl electrolyte.
2. The aspect ratio of the grooves machined via JEMM using the NaCl-EG electrolyte could be effectively enhanced by multiple passes. The depth, width, and aspect ratio of the grooves increased with an increasing feed depth and with more passes. However, an excessive feed depth and number of passes led to significant stray corrosion at the groove edges. Therefore, a feed depth of 20 µm and 10 passes were selected as the optimal parameters.
3. A square annular microstructure with a high geometric dimensional consistency and a high surface quality was achieved on Ti-6Al-4V vis JEMM with multiple passes using a NaCl-EG electrolyte. The width, depth, and surface roughness of the microstructure were 959 ± 3.4 µm, 119.8 ± 3 µm, and 0.539 ± 0.015 µm, respectively.

## Figures and Tables

**Table 1:** Properties of ethylene glycol.

Property	Parameter
Chemical structure	HOCH_2_CH_2_OH
Boiling point	197.85 °C
Density	1.1155 g/cm^3^
Melting point	−12.6 °C
Viscosity	0.02 Pa·s
Solubility of NaCl	5 wt% (room temperature)

**Table 2:** Main components of Ti-6Al-4V.

Element	Ti	V	Fe	Al	H	O	N	C
**Mass fraction**	89.335%	4%	0.3%	6%	0.015%	0.2%	0.05%	0.1%

**Table 3:** Parameters used for JEMM.

Machining Condition	Parameter
Material	Ti-6Al-4V
Inner diameter of nozzle	300 µm
Outer diameter of nozzle	600 µm
Electrolyte concentration	1 mol/L
Electrolyte temperature (°C)	25
Electrolyte pressure (MPa)	1
Machining gap (µm)	200
Applied voltage (V)	35
Feed rate (µm/s)	75

**Table 4:** Corrosion parameters evaluated from polarization curves of Ti-6Al-4V.

Sample	*E*_corr_ (mVSCE)	*J*_corr_ (µA cm^−2^)
NaCl-EG electrolyte	−409 ± 6	1.395 ± 0.064
aqueous NaCl electrolyte	−522 ± 7	4.716 ± 0.409

**Table 5:** Fitting parameters of EEC model for Ti-6Al-4V in different electrolytes.

Sample	*R*_s_ (Ω/cm^2^)	CPE-T (Ω^−1^s^P^cm^−2^)	CPE-P	*R*_ct_ (Ω/cm^2^)	W-R (Ω/cm^2^)	W-T	W-P
NaCl-EG electrolyte	98.76	2.4899 × 10^−5^	0.8712	11,717	191.9	0.01409	0.35247
aqueous NaCl electrolyte	1.048	8.5899 × 10^−6^	0.8261	329.7	728.1	0.33524	0.34574

## Data Availability

Data are contained within the article.
